# Genomic mechanisms underlying Bombay duck's rapid population surge

**DOI:** 10.1098/rspb.2026.1106

**Published:** 2026-11-04

**Authors:** Bin Kang, Linlong Wang, Dongji Yao, Jialin Geng, Zhicheng Sun, Rongfeng Cui

**Affiliations:** ^1^ Key Laboratory of Mariculture (Ministry of Education), Ocean University of China, Qingdao, Shandong, People's Republic of China; ^2^ Fisheries College, Ocean University of China, Qingdao, Shandong, People's Republic of China; ^3^ School of Ecology and State Key Laboratory of Biocontrol, Shenzhen Campus of Sun Yat-sen University, Guangdong, Shenzhen, People's Republic of China; ^4^ Southern Marine Science and Engineering Guangdong Laboratory - Zhuhai, Zhuhai, Guangdong, People's Republic of China

**Keywords:** fishery, population expansion, preadaptation, hypoxia

## Abstract

Bombay duck (*Harpadon nehereus*), once a minor by-catch in the East China Sea, has bloomed into a dominant species, altering marine ecosystems. This study explored the genomic basis of its mysterious recent success via chromosome-level genome assembly, comparative and population genomics. The genome assemblies of *H. nehereus* and its closely related outgroup species *Saurida undosquamis* were of good quality, showing a 96.7–96.8% Benchmarking Universal Single‐Copy Orthologues completeness. Population resequencing supports a single panmictic population throughout the coast of China. Expanded gene families and genes under intensified and positive selection are related to osmoregulation, hypoxia tolerance, immune function and reproduction. Recent selective sweeps are mostly related to immune response. These molecular signatures are consistent with long-term adaptations to a hypoxic, polluted and fluctuating environment. These findings suggest that species genomically preadapted to environments affected by global change can suddenly become new native invaders.

## 1. Introduction

Marine fish is one of the globally important protein sources, and its consumption increased at an average annual rate of 3.1% from 1961 to 2017, higher than the 2.1% rate of other animal protein foods (meat, dairy, milk, etc.), and almost twice the annual growth of the world's population (1.6%) for the same period [1]. The East China Sea possesses various habitats with high biodiversity, supporting an annual average catch of 4.44 million tons, accounting for approximately 10% of the global catch from large marine ecosystems [2]. However, owing to overfishing and habitat destruction, fisheries in the East China Sea have experienced decades of decline and a shift in catch composition from high trophic large species to small pelagic species [3].

Bombay duck (BD, *Harpadon nehereus*) belongs to the family Synodontidae of the order Aulopiformes. Aulopiform fishes are widely distributed in both deep-sea and coastal areas, showing sophisticated evolutionary adaptations, e.g. bioluminescence, thin bones, tubular or greatly reduced eyes, hermaphroditism and large mouths with dagger-like teeth [4]. BD was first recorded by Hamilton (1822), who named it *Osmerus nehereus*, according to the specimen from the Ganges estuary. Later, Günther [5] assigned it to *H. nehereus* and postulated its deep-sea origin, supported by its extremely close resemblance to deep-sea fish in morphology, including gelatinous consistency, a large mouth with a loosely articulated lower jaw armed with recurved teeth and colour as phosphorescent and semi-translucent milky white [6]. These traits differ significantly from its closely related outgroup species, brushtooth lizardfish (BL, *Saurida undosquamis*), which is characterized by normal water content, robust muscles, fully developed scales, lack of recurved teeth and strong pigmentation. Surprisingly, BD does not inhabit the deep sea. Instead, it is distributed in year-round oxygen-minimum zones [7] of shallow waters, such as the Gulf of Cambay [6], the Bay of Bengal [8] and the Ganges estuary [9], accounting for about 10% of the estimated average annual marine fish landings of India [10] and above 10% of the total marine catch of Bangladesh over the past decades [11]. BD had traditionally been a minor by-catch, whose landings were too low to be recorded in the official statistics in the People's Republic of China. This species suddenly bloomed in 2005 by contributing to 83% of the total catch in the main fishing grounds in the East China Sea [12], and it has repeatedly bloomed in later years [13–15]. Increasing from a minor population to the dominant population, BD is a new ‘invader’ because it has brought ecological impacts by breaking the balance of community composition through competing for food resources with other species [16]. In this context, we define invaders as species imposing deleterious impacts on the ecosystem, independent of their geographical origin.

The biomass increase of *H. nehereus* (BD) is a result of the synergistic interaction between its unique physiological and ecological traits and external anthropogenic and environmental disturbances. Physiologically, its exceptional hypoxia tolerance and efficient metabolic regulation [16,17] provide a critical adaptive advantage to monopolize niches uninhabitable by most commercial fish. Ecologically, its r-selected reproductive strategy, including early maturity, high fecundity and short lifespan, allows it to quickly fill the demersal niche vacated by overfished traditional species [6]. Extrinsically, overfishing has depleted top predators and competitor species, releasing ecological niches [3], while climate change exacerbates hypoxia by reducing discharge from dammed rivers or increasing discharge with excessive nutrients and pollutants [18].

So far, the genetic bases that facilitate the recent BD blooms in the East China Sea are unclear. Pre-existing genomic adaptations may have left detectable signatures within the BD genome related to its recent success. In this study, we first assemble and annotate chromosome-level reference genomes for BD and its closely related outgroup species BL (*S. undosquamis*). We then test for expanded and contracted gene families along the BD branch. Next, we screen for genes under intensified, relaxed and positive selection on the BD branch. Finally, we use population whole-genome resequencing data to test for geographical isolation, as well as recent selective sweeps in BD populations. Our results show that molecular evolutionary signatures in the BD genome are consistent with the known phenotypes that facilitate their recent bloom.

## 2. Material and methods

### (a) Ethics statement and sample collection

All animal experimental procedures in this study were approved by the Animal Care and Ethics Committee of the Ocean University of China with the protocol ID OUC-AE-2019-047.

The adult BD was wild-caught in the East China Sea in October 2019 (supplementary material, figure S1). Meanwhile, another lizardfish, BL (*S. undosquamis*), was collected from the Beibu Gulf of the South China Sea in May 2022 and included in the analysis as an outgroup for comparison (supplementary material, figure S2). The sex of these specimens was unidentified. Although closely related, BL is a resident species in coastal shallow waters, lacking specialized morphologies compared to BD and does not exhibit blooming. The muscle tissues of adult BD and BL were harvested for DNA extraction, genome sequencing and high-throughput chromosome conformation capture (Hi-C) library construction, while the heart, eye, brain, gill, kidney, spleen, liver, head kidney, gonad, stomach and intestine were collected for RNA sequencing to provide comprehensive transcripts for gene prediction of the assembled genomes. All tissues were snap-frozen using liquid nitrogen over 24 h and stored at –80°C before sample preparation. Tissues were exhausted for sampling.

### (b) Genome assembly

Details of nucleic acid extraction, genome assembly and annotation are given in supplementary methods.

### (c) Comparative genomic analysis

The orthologous genes among 11 species (BD, BL, *Chaenocephalus aceratus*, *C. harengus*, *Danio rerio*, *Gasterosteus aculeatus*, *Hippocampus comes*, *Lepisosteus oculatus*, *Liparis tanakae*, *Pseudoliparis swirei* and *Takifugu rubripes*) were detected by OrthoFinder (version 2.3.3) [19]. Their single-copy orthologous genes were aligned with MUSCLE (http://www.drive5.com/muscle/) [20] and then concatenated into a multiple sequence alignment matrix, as input into RAxML [21] for maximum-likelihood (ML) phylogenetic tree construction. The resulting ML tree and fourfold degenerate site alignments were used to estimate divergence time by the MCMCTree program of PAML (version 4.9) (http://www.abacus.gene.ucl.ac.uk/software/paml.html) [22]. Seven calibration points were selected from the TimeTree database (http://www.timetree.org/) [23], including *Danio*–*Clupea* divergence at 46–151 Mya, Otomorpha–Euteleosteomorpha at 180–264 Mya, Aulopiformes–*Hippocampus* at 146–182 Mya, *Hippocampus*–*Takifugu* at 96–122 Mya, *Takifugu*–*Gasterosteus* at 82–174 Mya, *Chaenocephalus*–*Gasterosteus* at 53–134 Mya and *Gasterosteus*–*Liparis* at 40–91 Mya.

To identify gene family dynamics on the branch leading to BD, the gene family size differences between the ancestor and each species along the ML phylogenetic tree were estimated by CAFÉ5 software (http://www.sourceforge.net/projects/cafehahnlab/) [24]. A *p*-value threshold of 0.05 was used to identify gene families that exhibited significant expansion or contraction along each branch of the ML phylogenetic tree.

### (d) Positive selection, intensified and relaxed selection tests

To investigate whether the protein-coding genes underwent intensified or relaxed selection at a macro-evolutionary timescale, BUSTED-MH [25] implemented in HYPHY version 2.5.10 and RELAX [26] were applied to test positive selection and relaxed selection in BD, respectively. Single-copy orthologues were identified by running Genespace on the longest isoforms of genes of the 11 species in 2.7. Each gene was aligned with TrimAl to remove regions of poor alignment quality. The BD branch was marked as the foreground while all other branches served as the background.

### (e) Genomic resequencing and single nucleotide polymorphisms calling

In addition to the reference individual, we broadly sampled from the known distribution regions of BD along the coast of China in October 2019 at depths of approximately 20 m. A total of 48 individuals of unknown sex from six populations were sampled for genomic resequencing, including Qingdao (QD, *n* = 2), Lianyungang (LYG, *n* = 10), Zhoushan (ZS, *n* = 10), Wenzhou (WZ, *n* = 8), Yangjiang (YJ, *n* = 8) and Lingao (LG, *n* = 10), from north to south. These include the geographical boundaries of the distribution of BD along the Chinese coast (QD and LG), as well as the populations that have experienced significant expansion (ZS and WZ).

Genomic DNA was extracted from the muscle tissues and sequenced by Illumina HiSeq 2500. All samples were exhausted. After quality control, high-quality reads were aligned against the BD genome using BWA-MEM. The resulting alignments were processed following the GATK Best Practices. Specifically, SAMtools (v1.16) was used for coordinate sorting and indexing. To account for library artefacts, read group information was added, and PCR duplicates were identified and removed using GATK (v4.2.1.0) [27] MarkDuplicates. Variant calling was performed using the GATK HaplotypeCaller in GVCF mode for each individual, with a minimum mapping quality of 30 and a maximum of 250 reads per alignment start to ensure reliability. Subsequently, joint genotyping was executed via GenotypeGVCFs to produce the raw VCF file.

### (f) Population structure and historical demographic analysis

A principal component analysis (PCA) was performed at the individual scale for the six BD populations using GCTA v1.24.2 [28]. With input generated by Plink [29], the population genetic structure was inferred using Admixture [30], with the number of genetic clusters (*K*) predefined from two to six. Furthermore, an individual-based (neighbour-joining tree) (NJ tree) was constructed based on the *p*-distance using TreeBeST v1.9.2 (http://treesoft.sourceforge.net/treebest.shtml).

The ancient demographic history of BD was inferred using a hidden Markov model approach as implemented in pairwise sequentially Markovian coalescent (PSMC) [31] based on single nucleotide polymorphism frequencies. An average mutation rate of 2.74424 × 10⁻⁹ per base per generation (estimated in 2.7) and a generation time of 1 year [6] were used for the analysis.

### (g) Kinship coefficient and runs of homozygosity analysis

To measure the relatedness between individuals, we estimated the coefficient of kinship [32] and the probability of identity by descent using the popkin R package [33]. To further clarify whether the expansion of BD's population relies on inbreeding between closely related individuals, we inferred runs of homozygosity (ROH) and the inbreeding coefficient *F*_ROH_ of each individual with the software ROHan [34]. Subsequently, we calculated the mean ROH value for each population by averaging the ROH of the individuals within each group.

### (h) Detecting recent selective sweeps in the Bombay duck genome

A convolutional neural network approach implemented in diploS/HIC [35] was used to identify genomic regions under selective sweeps in the BD population. We first performed population genetic simulations under different types (hard, soft and neutral) of selective sweeps by Discoal using the demography inferred by PSMC (N0 = 97344.56, Pt = 117.5402–352.6206 and Pre = 261.8726–2618.726; see supplementary material for complete command lines). DiploS/HIC was first trained and tested using simulated population genetic data. The resulting trained model was then applied to make predictions on empirical data. As recommended by the user manual, the simulations were performed on a genomic segment of 55 kb, composed of eleven 5-kb windows. The final diploS/HIC was predicted in 5-kb windows across the genome, and each window was classified as neutral, soft, hard, softLinked or hardLinked. We treated windows with low probability (*p* < 1 × 10^−6^) of neutrality as candidate regions under recent positive selection.

### (i) Annotating the deleterious variants in the Bombay duck population

Variant impact was annotated by SnpEff version 5.1d [36]. The population-specific synonymous and non-synonymous sites, with respect to the reference genome, were used to evaluate their functional impacts, which are classified as low, moderate and high. SNPs with a frequency below 5% or above 95% were excluded for this analysis. The variants were polarized by the outgroup species BL and *Lampris incognitus*. The alleles shared by both outgroups were defined as ancestral, while sites where outgroups were inconsistent were excluded. Alleles differing from the ancestral state were identified as derived and deemed potentially deleterious.

## 3. Results

### (a) Genome assemblies and annotation

The estimated genome size of BD was 1298.2 megabase (Mb) based on the SGA-preqc method (supplementary material, table S1 and figure S3). After removing haplotigs and contig overlaps, the PACBio assembly length was 1357.87 Mb with 735 contigs of an N50 of 5.66 Mb (table 1). Based on the interaction matrix from 159.28 Gb Hi-C data, these contigs were scaffolded into 24 pseudochromosomes (supplementary material, figure S4 and table S2), which anchored 97.34% of the bases contained in assembled contigs. The scaffold N50 of the final chromosome assembly reached 55.20 Mb (table 1). BUSCO analysis revealed that 96.80% of complete genes were detected in the BD genome assembly (supplementary material, table S3). A total of 586.99 Mb was identified as repeat elements in the BD genome, accounting for 43.23% of the total genome size and 8.74% was identified as tandem repeats (supplementary material, table S4). For TEs detection, approximately 12.10% of the genome was retrotransposons, of which long terminal repeats contributed most (7.60%). The repeat-masked genome was used for gene prediction and genome annotation, resulting in 44 395 protein-coding genes (figure 1A).

**Table 1.** Genome assemblies of BD (*H. nehereus*) and BL (*S. undosquamis*).

	BD		BL	
sample ID	contig	scaffold	contig	scaffold
total	1 357 813 686	1 357 870386	1 319 851 155	1 319 878 255
number	735	218	325	78
N25	12 206 029	58 252 852	36 169 110	58 830 462
N50	5 664 681	55 201 974	19 160 715	53 644 477
N75	2 471 643	51 418 773	6 487 716	50 115 062
gene number	30 885		32 333	
BUSCO (%)	96.8		96.7	
total repeat content (%)	43.17		45.29

**Figure 1.**
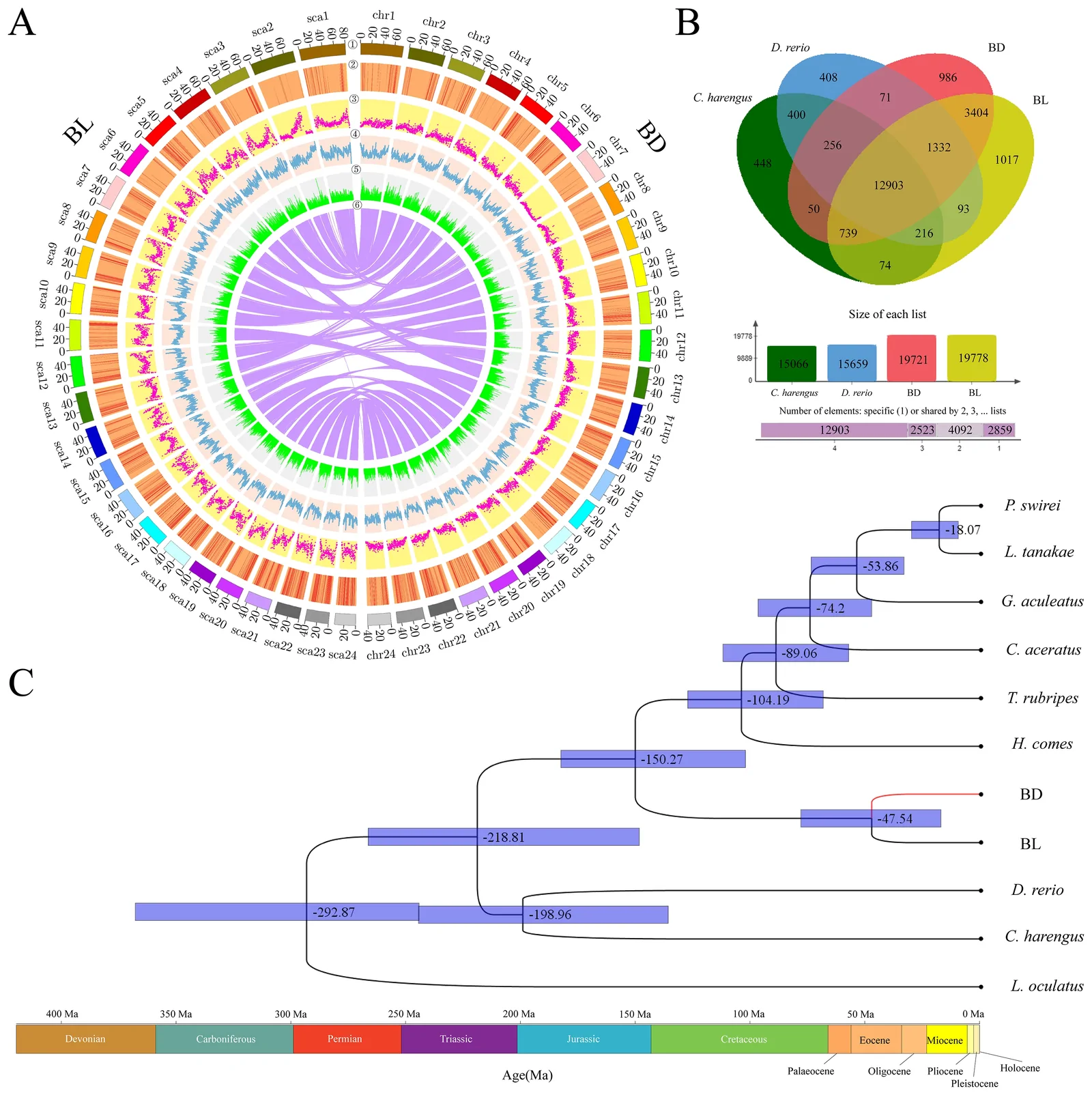
(A) Genome characteristics of BD and BL, (B) statistics of gene family clusters and (C) a phylogenetic tree with a divergence time. From the outer circle to the inner circle: 1, chromosome; 2, GC content; 3, heterozygosity; 4, repeat density; 5, gene density; 6, chromosome synteny.

For the outgroup BL, the estimated genome size was 1198.5 Mb (supplementary material, figure S3). Moreover, 74.15 Gb Oxford Nanopore long-read sequencing data (supplementary material, table S1) were used to construct an assembly consisted of 325 contigs with an N50 of 19.16 Mb. Finally, the contigs were scaffolded into 24 pseudochromosomes (supplementary material, figure S4 and table S2). BUSCO analysis revealed that 96.70% complete genes were detected in the BL genome assembly (supplementary material, table S3). In the BL genome assembly, a total of 611.27 Mb accounting for 46.32% of the genome was identified as repeat elements (supplementary material, table S4), and 42 089 protein-coding genes were annotated for subsequent analysis (figure 1A).

These high-quality assemblies provided a robust genomic foundation for investigating the evolutionary mechanisms underlying the ecological success of BD.

### (b) Phylogeny, divergence times and population dynamics

We used 2863 single-copy genes to reconstruct a phylogeny (figure 1B; supplementary material, figure S5 and table S5), which had full support on all nodes. The results confirmed the monophyly of BD and BL, which shared a common ancestor about 47.5 million years ago (figure 1C). To understand the current status of BD's population, we further analysed the genetic structure and demographic history of 48 individuals across its distribution range.

Population resequencing of 48 individuals from six sampling sites (figure 2A) yielded 771 Gb of genomic data at a total of 480 × effective sequence depth for the subsequent analyses (supplementary material, table S11). We identified 3 432 037 high-quality SNPs, including 1 580 899 in intronic regions, 1 567 322 in intergenic regions and 161 690 in exonic regions (supplementary material, table S12). Except for the QD population, the nucleotide diversity (π) of the other five populations ranges from 5.09 × 10^–4^ to 5.55 × 10^–4^, while that of the QD population was slightly smaller (3.64 × 10^–4^) (figure 2B; supplementary material, figure S6A). The results of Admixture and NJ trees were consistent with PCA (figure 2C). Regardless of the choice of *K* (the number of source populations), BD samples are not clustered by their geographical locality (figure 2D). The lowest *k* = 2 had the smallest CV error (supplementary material, figure S6B). Even at this *k*, the populations did not segregate by locality, suggesting a panmictic population. The NJ tree based on SNPs supported the results of Admixture, which showed that individuals from different localities did not reveal distinct gene pools among sampled locations (figure 2E).

**Figure 2.**
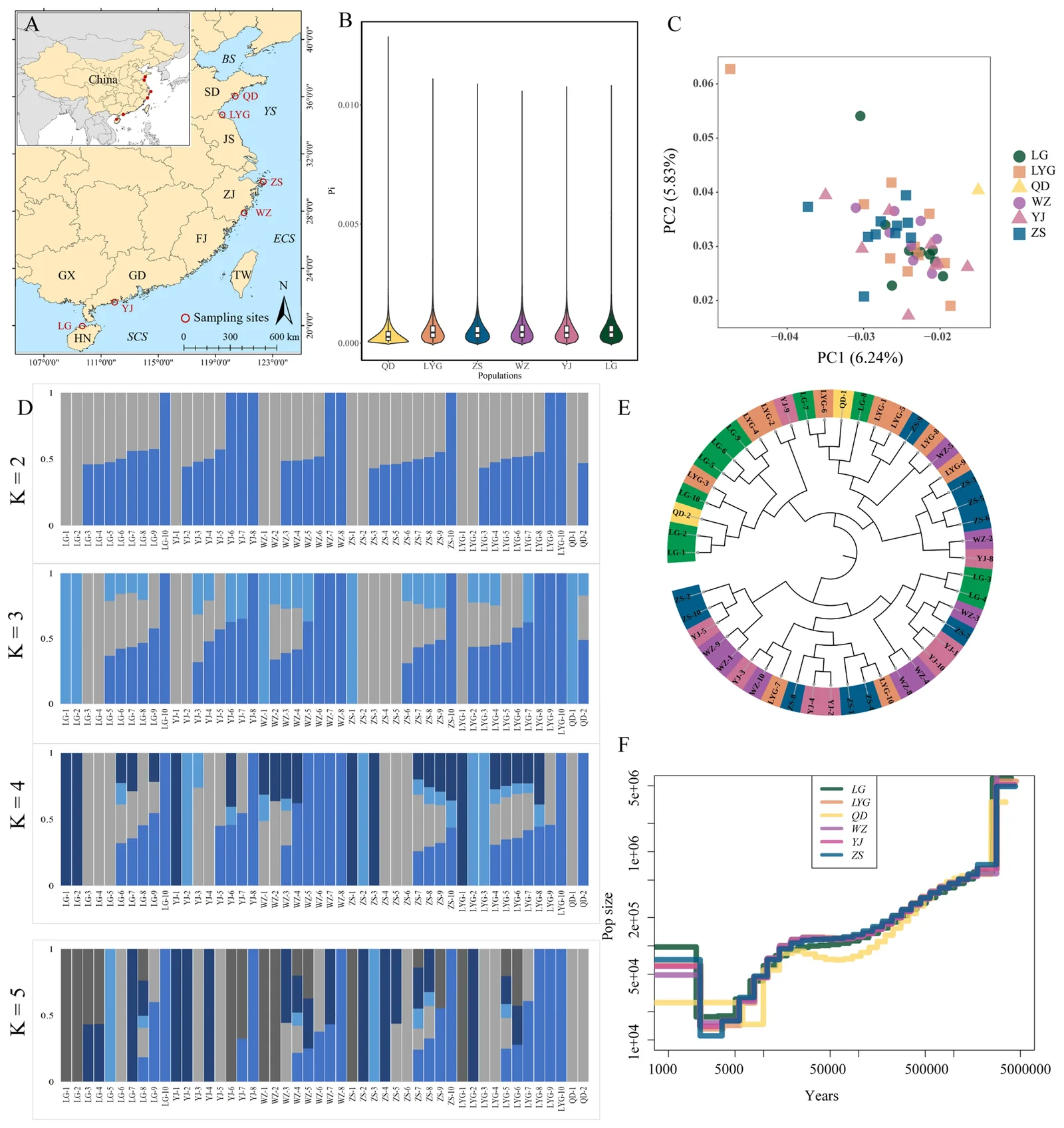
(A) The map of sampling sites, (B) genome-wide distribution of nucleotide diversity (π) in 10-kb windows with 5-kb increments, (C) the first two principal components of a PCA based on whole-genome SNPs, (D) admixture analysis of six BD populations (*K* = 2–5), (E) neighbour-joining tree constructed using *p*-distances of 48 individuals and (F) demographic history of the BD populations.

The PSMC analysis showed that different populations of BD had similar population historical dynamics. Assuming a generation time of 1 yr^–1^, BD experienced a continuous decline in population size about 10 million to 5000 years ago, and displayed obvious population expansions about 2000 years ago (figure 2F).

The pairwise relatedness coefficients and frequency statistics between BD individuals showed that the relatedness was low, mostly close to zero (supplementary material, figure S7), suggesting outbreeding. Accordingly, the proportion of the runs of homozygosity in each population was low, ranging from 0.4% (QD population) to 1.4% (ZS population) (supplementary material, figure S8), confirming the outbreeding nature of these samples.

The high genetic connectivity and historical expansion potential set the stage for exploring the specific genomic adaptations that fuelled its ecological success.

### (c) Gene family dynamics

Among 14 543 gene families across 11 teleost species (table 2; supplementary material, table S6a), BD had 789 expanded (*p* < 0.05: 39, FDR ≤ 0.2: 14) and 576 contracted (*p* < 0.05: 26, FDR ≤ 0.2: 2) gene families. The expanded gene families (FDR ≤ 0.2) were significantly enriched in Gene Ontology (GO) terms such as optic nerve formation (*p* = 1.56 × 10−^42^), cytoskeleton-dependent intracellular transport (*p* = 1.65 × 10^−34^), glial cell development (*p* = 4.32E-30), ultradian rhythm (*p* = 3.06 × 10^−28^), regulation of intracellular oestrogen receptor signalling pathway (*p* = 9.00E-27), adult locomotory behaviour (*p* = 3.12E-17), long-chain fatty acid metabolic process (*p* = 5.28E-15), sperm capacitation (*p* = 7.32E-17) and regulation of growth (*p* = 1.05E-14). In addition, the gene families related to humoral immunity also expanded, such as cellular response to interleukin-4 (*p* = 1.71e-33), positive regulation of T-cell-mediated cytotoxicity directed against tumour cell target (*p* = 3.25E-23, figure 3A; supplementary material, table S6b). Notably, although the GO term cellular response to hypoxia was not significantly enriched (*p* = 0.11), the Endothelial PAS domain-containing protein 1 (*epas1*) involved in hypoxia tolerance was expanded into three copies (lty026669-T1/lty035875-T1/lty047576-T1) in the BD genome, whereas only a single copy was identified in the outgroup BL and other teleosts. Other GO terms related to hypoxia tolerance, such as blood vessel remodelling (*p* = 0.0057), regulation of heart rate (*p* = 0.0066) and haematopoietic stem cell differentiation (*p* = 0.0185), were significantly enriched. The contracted gene family (OG0000028, major histocompatibility complex class I) was enriched for a single GO term immune response (*p* = 7.84E-8). Compared to the outgroup BL which had 26 paralogues, BD only contained four paralogues in this family.

**Table 2.** Summary of genomic signatures of molecular evolution in BD *Harpadon nehereus*.

	type	number	significance threshold
gene families	expanded	789 (14 significant)	FDR ≤ 0.2
	contracted	576 (2 significant)	FDR ≤ 0.2
orthologous genes	relaxed	920	FDR < 0.05
	intensified	2546	FDR < 0.05
	positive selection	93	*p* < 0.05
population	hard/soft sweeps	41	*p* < 1 × 10^−6^ (DiploSHIC)

**Figure 3.**
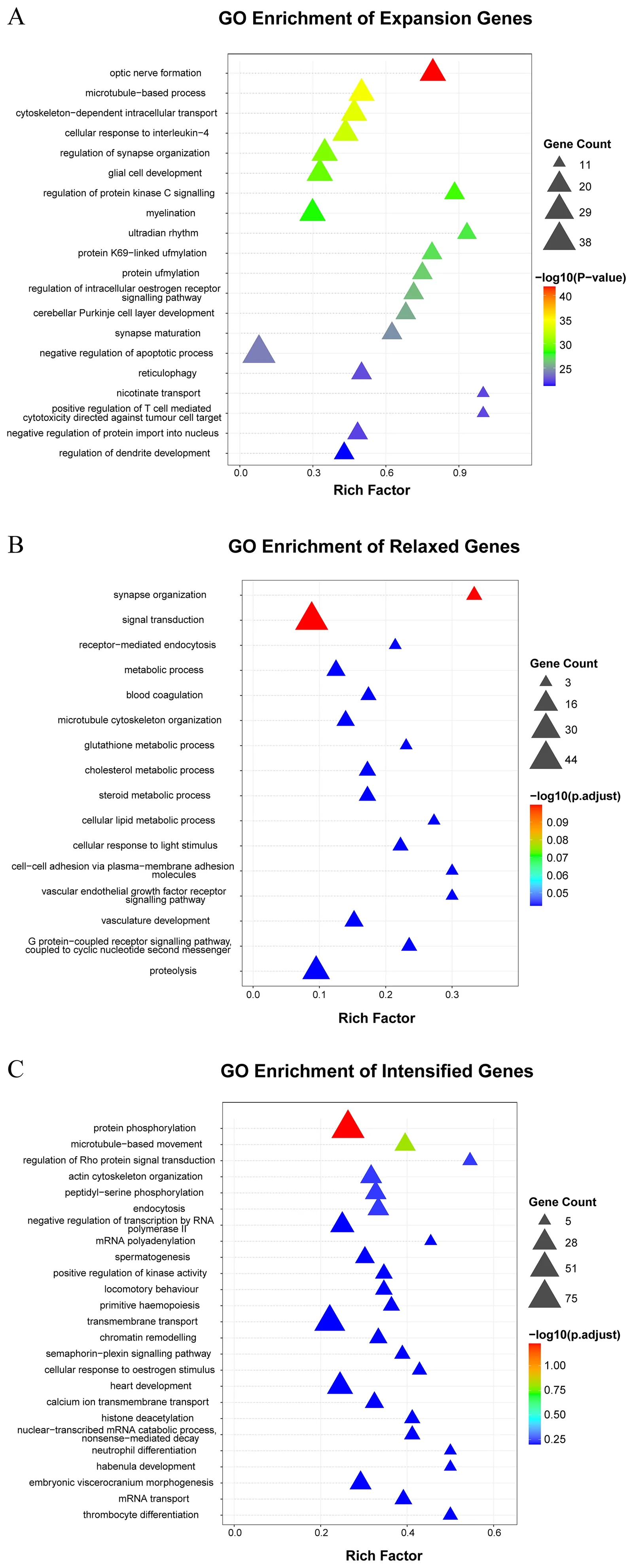
GO enrichment analysis of expanded gene families, relaxed genes and intensified genes in the BD (*Harpadon nehereus*). (A) Expanded gene families, (B) relaxed genes and (C) intensified genes in the BD genome.

### (d) Genes under relaxed and intensified selection in Bombay duck

A total of 920 genes of BD was under relaxed selection (FDR < 0.05, table 2; supplementary material, table S7a), which were significantly associated with functions such as signal transduction (*p* = 0.0031), synapse organization (*p* = 0.0038), proteolysis (*p* = 0.009), vasculature development (*p* = 0.0157), steroid metabolic process (*p* = 0.024) and blood coagulation (*p* = 0.041). In addition, relaxed genes were significantly related to metabolic process (figure 3B; supplementary material, table S7b).

BD showed more genes under intensified selection (2546 genes with FDR < 0.05; supplementary material, table S8a) than under relaxed selection. Intensified genes were significantly enriched in GO terms such as protein phosphorylation (*p* = 9.98e-05), microtubule-based movement (*p* = 0.00053), endocytosis (*p* = 0.0036), cellular response to oestrogen stimulus (*p* = 0.024), embryonic cranial skeleton morphogenesis (*p* = 0.013), embryonic viscerocranium morphogenesis (*p* = 0.013), endocytosis (*p* = 0.002), heart development (*p* = 0.021), primitive haemopoiesis (*p* = 0.027), locomotory behaviour (*p* = 0.027), spermatogenesis (*p* = 0.028) and ion transport (*p* = 0.04). We found the genes under intensified selection were also related to immune response, like neutrophil differentiation (*p* = 0.019) and toll-like receptor signalling pathway (*p* = 0.043, figure 3C; supplementary material, table S8).

### (e) Positive selection analysis

We identified 93 positively selected genes in BD (table 2; supplementary material, table S9a), which were enriched for GO functions such as nucleocytoplasmic transport (*p* = 0.024), mitochondrial fusion (*p* = 0.024), DNA damage checkpoint signalling (*p* = 0.027), neutrophil differentiation (*p* = 0.029), thymus development (*p* = 0.031), haem biosynthetic process (*p* = 0.034) and endoderm formation (*p* = 0.039). Notably, 34 positively selected genes were also confirmed by RELAX to be under intensified selection (supplementary material, table S10). Among them, *ephb1* and *hecw2* are involved in neurogenesis and angiogenesis. The *cep290* gene is involved in early and late steps in cilia formation. The *aifm1* gene plays an important role in the regulation of respiratory chain biogenesis and serves as a proapoptotic factor in response to apoptotic stimuli. The gene *ino80* is involved in DNA repair. *Hoxd10a* may be involved in fin development. The *nrl* gene regulates photoreceptor development. The *mitofusin-1* gene is related to vascular remodelling under hypoxia [37]. Upregulation of *prex1* during ageing helps maintain naïve CD4 + T-cell proliferation at the periphery [38]. The *greb1* gene regulates the response to oestrogen and plays an important role in hormone-responsive tissues.

### (f) Genome-wide selection pressure analysis

Annotation of the polymorphism of SNPs in the BD population showed that the mutations with high deleterious potential generally segregated at low frequencies, suggesting effective purifying selection (supplementary material, figure S9).

Population genetic data could reveal positive selection at a much more recent timescale than divergence data. DiploSHIC identified 41 genes to have experienced selective sweeps, reflecting recent adaptive evolution. These genes encompass several critical functions, including *ttn* related to cardiac function and development (table 2; supplementary material, table S13 and figure S10), *bgal* and *lcp2* associated with immunity, *cimpr* and *zbtb16a* linked to immune stress response.

## 4. Discussion

Being a vast interconnected ecosystem, the ocean imposes limited biogeographic barriers to fish. Rare immigrants usually do not pose threats to local ecosystems. Nevertheless, if a migratory species abruptly expands to become a dominant species in a local ecosystem, it would bring ecological impacts by breaking the balance of community composition and altering the ecosystem services as an ‘ecosystem engineer’ and could be regarded as a new ‘invader’ [39].

Rapid recent population expansion of BD requires the ability to respond freely to large changes in environmental factors such as dissolved oxygen and water depth [16], a strong immune system to protect itself from various microorganisms in different environmental conditions [17], and multiple reproductive cycles (up to six) per year [6]. The genome resources and the population genomics data will lay the foundation for understanding the unexpected BD bloom in the coastal community.

### (a) Population structure and demographic history

The BD samples collected from a broad geographical distance belong to a single population genetically, which indicates frequent gene flow, a very large effective population size and a lack of strong barriers to dispersal. This result is consistent with previous studies based on genotyping by sequencing (GBS) and mitochondrial markers. For example, based on GBS, five geographical populations (QD, Nantong, ZS, Chaozhou and Zhanjiang) of BD [40] show no population structure. The cytochrome *b* sequences of seven populations (QD, Nantong, ZS, Sanmen, Ningde, Quanzhou and Zhanjiang) suggest intra-population instead of inter-population variance as the main contributor to their sequence variations [41]. Marine fishes are known to have limited population structure owing to their high dispersal potential at various stages of the life cycle [42]. Ocean current systems can carry marine life hundreds of kilometres away [43,44]. BD has the habit of inshore breeding [6]. The planktonic eggs and larvae can disperse along the complex current systems in the northwest Pacific Ocean, such as the Taiwan Warm Current, the South China Sea Warm Current and the China Longshore Current, which may be one of the main reasons for promoting gene exchange in coastal waters of China. Similar results were also presented in population genetic analyses on other species, such as *Anguilla Japonica* [45], *Larimichthys polyactis* [46] and *Sebastiscus marmoratus* [47].

PSMC analysis showed that the effective population size of BD decreased sharply during the Pleistocene glaciation from about 70 to approximately 11.5 kya, probably owing to the dramatic climate changes such as lower temperatures and sea levels [48], leading to the isolation of populations [49]. Meanwhile, the penultimate glacial period (30 000–130 000 years ago) of the Pleistocene [50] may also correlate with a decline in population. Therefore, changes in the historical population size of BD were mainly related to the alternation between glacial periods (lower temperature, lower sea level and habitat contraction) and interglacial periods (warmer climate, higher sea level and habitat expansion) in the Pleistocene.

### (b) Potential links between molecular evolution signatures and Bombay duck phenotypes

Research on the biology and ecology of BD has been increasing with its economic status, and the molecular mechanisms of its special traits have attracted researchers’ attention. The high-quality BD and BL genomes and annotations lay the foundation for future studies linking phenotypes to genotypes.

The morphology of BD resembles that of deep-sea fish by having poorly mineralized bones [51] and gelatinous muscles, leading to a previous hypothesis by Günther [5] to speculate its deep-sea origin. However, we do not observe an enrichment of the GO terms related to calcium ion transport in the expanded, relaxed, intensified or positively selected genes. Most of the genes found significant in these tests (e.g. *cacna1aa*, *cacnb2, slc8a1*, etc.) are related to the generation of action potential in the nervous, muscular or cardiovascular system, as opposed to influencing bone formation [52–54]. Therefore, remodelling of these calcium ion transport genes may not be the direct cause of the poor mineralization of bones, but may be an evolutionary response to a deficiency of calcium ions. Moreover, the high water content of BD reaching 90% and the high permeability of its thin skin [16] may reduce sodium ion concentrations in the body fluid. Accordingly, intensified genes are enriched for the GO term ion transport, suggesting potential remodelling in response to higher water content [55]. Among them, the intensified gene *slc9a1b* may be related to maintaining the intracellular neutral pH [56] and cell volume, while *slc9a3* is a major apical Na^+^/H^+^ exchanger in the kidney and intestine, playing an important role in renal and intestinal Na^+^ absorption and blood pressure regulation [57]. In addition, expanded genes were enriched for the GO term transmembrane transport. This is driven by the expansion of the *slc22a13b* gene into 11 paralogues in BD. *slc22a13* genes are mainly involved in organic cation transportation across the cell membrane, driving urate and nicotinate renal reabsorption. Osmoregulation in elasmobranchs is achieved by retaining a high concentration of urea in the blood by reabsorption [58]. It would be interesting to explore in future studies whether BD evolved a similar strategy accompanying the increase in water content.

Globally, anoxic zones in coastal waters have grown exponentially since the 1960s [59]. The decrease in oxygen solubility in ocean water under climate warming and the inhibition of oxygen diffusion by increasingly stable ocean stratification disrupt biological control mechanisms, resulting in changes in species distribution, mass individual mortality and loss of biodiversity [60]. The East China Sea is a major upwelling zone and contains one of the largest near-shore hypoxic zones in the world's oceans [7]. Preadaptation to hypoxic environments may have facilitated the boom of BD in recent years. Indeed, gene families associated with blood vessel remodelling expanded in the BD genome. *epas1* expanded into three copies, potentially promoting the oxygen transport capacity of erythrocytes to improve the ability to adapt to low-oxygen environments, affecting the haematopoietic ability of BD [61]. Genes under intensified selection are enriched for heart development and primitive haemopoiesis. For example, forkhead box protein O3 (*foxo3*) has been confirmed to play a key role in alleviating hypoxic damage in fish [62,63] and is a well-known gene in life-span regulation; *gata1* is involved in erythroid differentiation; *ngb* inhibits cellular respiration in response to hypoxia. Genes under positive selection in BD are enriched for the GO term haem biosynthetic process. The gene *nipbl* is critical for haematopoiesis by regulating *RUNX1* [64]. Capillaries are stimulated to regenerate in some fish species, thereby enhancing their oxygen-carrying capacity in low-oxygen environments [65,66]. In BD, the positively selected gene *ephb1* may effectively promote angiogenesis through interaction with its ligand Ephrin-B2 [67]. The positively selected integrin *itga4* represses PRKCA-mediated L-type voltage-gated channel Ca^2+^ influx and ROCK-mediated calcium sensitivity in vascular smooth muscle cells via its interaction with *SVEP1*, thereby inhibiting vaso-constriction [68].

Multiple rounds of reproduction per year may underlie the rapid recent expansion of the BD population size. Interestingly, expanded gene families are highly enriched for the GO term regulation of the intracellular oestrogen receptor signalling pathway. We also identify the gene *greb1* as both positively selected and under intensified selection. *greb1* regulates oestrogen receptor expression, and its expression is correlated with the female ovulation cycle [69]. The GO term sperm capacitation is significantly enriched in expanded gene families. Intensified genes were also enriched for the function of spermatogenesis. Overall, molecular evolution signatures are consistent with heightened selective pressure on both female and male reproductive functions in BD.

Seawater eutrophication in coastal China has led to a surge in pathogenic bacteria causing diseases like red skin and visceral leukoderma [70–75] by pathogens like *Pseudomonas* and *Vibrio*, posing severe health risks to BD. These environmental stresses should impose selective pressure on BD in genes related to immune functions. Specifically, the positively selected interleukin receptor 7 (*il7r*) and genes involved in lymphatic development (*prox1a* and *lcp2b*) can facilitate T-cell maturation and immune signalling [76–82]. The positive selection of *ephx4* likely improves detoxification [83], while *ncor1* regulates B-cell development [84]. The significant positive selection on genes involved in immune function probably strengthens BD's ability to recognize and eliminate exogenous pathogens, providing a crucial competitive advantage in bacterially dense habitats [85,86].

We caution that while comparative and population genomics are valuable in revealing the potential molecular bases of specialized phenotypes, future functional experiments will be required to validate the candidates identified in this study.

## 5. Conclusions

A high-quality and chromosome-level genome of BD was assembled and annotated, providing insights into its genomic characteristics and ecological adaptations in evolutionary processes. BD contains expanded gene families, intensified and positively selected genes closely related to its observed morphological and physiological traits such as osmoregulation, hypoxia tolerance and enhanced immunity. Population genomics revealed a single, panmictic large population that experiences efficient natural selection. The molecular evolution signatures of the BD genome are consistent with the traits that facilitate BD's rise in the East China Sea. The example of BD shows that global change may disrupt established marine communities by allowing preadapted potential invaders to quickly expand.

## Supplementary Material



## Data Availability

The data generated in this study have been deposited in public databases. The BD genome assembly and population resequencing data are available in the China National GeneBank DataBase (CNGBdb) CNSA under the accession number CNP0007843. The BL genome assembly and raw data are deposited in CNGBdb CNSA under the accession number CNP0007838. Additionally, sequencing data related to the BD genome assembly have been deposited in the National Center for Biotechnology Information (NCBI) BioProject database under the following accession numbers: PRJNA784345 (Hi-C data), PRJNA784315 (NGS data) and PRJNA784648 (PacBio HiFi data). Direct URLs to these datasets are listed in tables S1 and S3 in the supplementary material. Custom code has been uploaded to Zenodo and a Github release https://github.com/bc2070/Harpadon_nehereus/releases/tag/v1.1.4. [87]. Supplementary material is available online [88].
