# Supplementary material for: Genomic mechanisms underlying Bombay duck's rapid population surge
Source: Proc Biol Sci. 2026 Nov 4;293(2082):20261106. doi: 10.1098/rspb.2026.1106 (PMC13730804; doi:10.1098/rspb.2026.1106)
Supplement: Supplementary file 1 [file rspb.2026.1106.zip › rspb.2026.1106/rspb20261106_si_001.pdf]

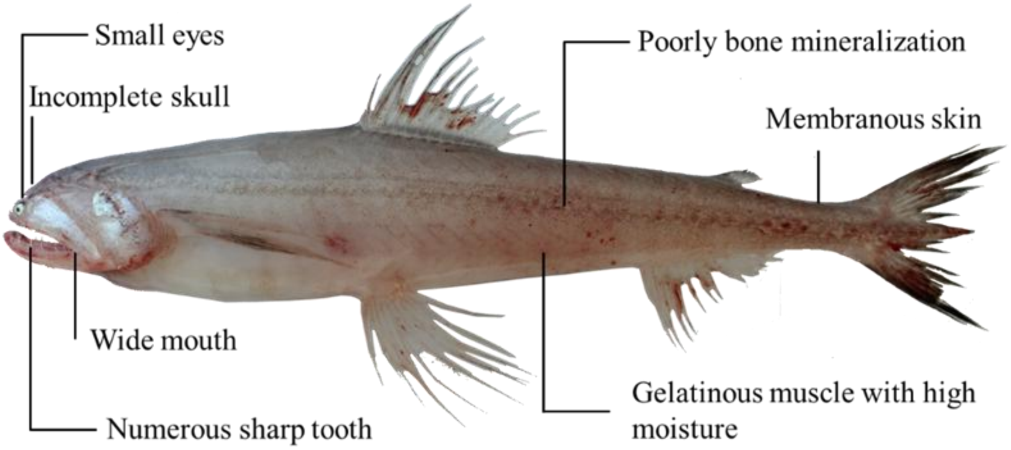

Figure S1 Phenotypic characteristics of Bombay duck *Harpadon nehereus*.

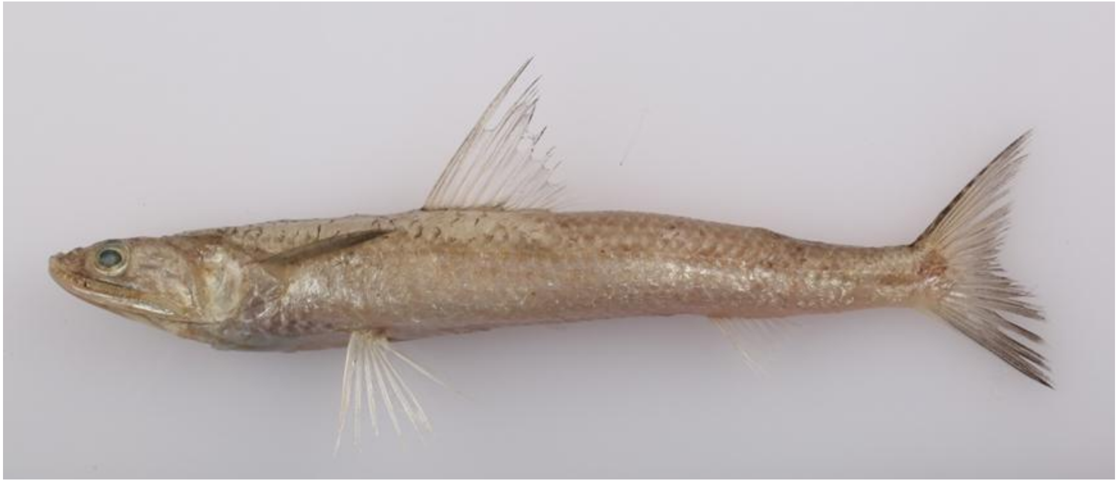

Figure S2. Photo of Brushtooth lizardfish *Saurida undosquamis*.

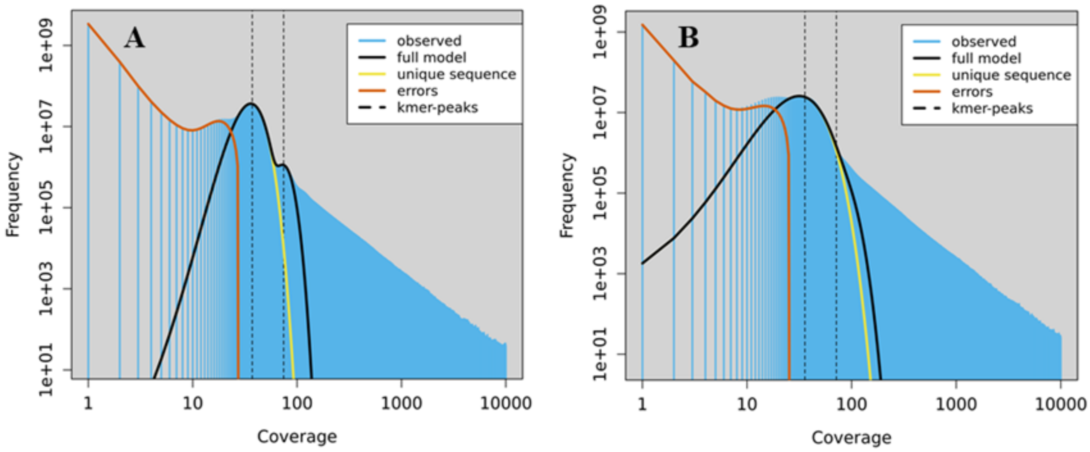

Figure S3 GenomeScope profiles for *Harpadon nehereus* (A) and *Saurida undosquamis* (B).

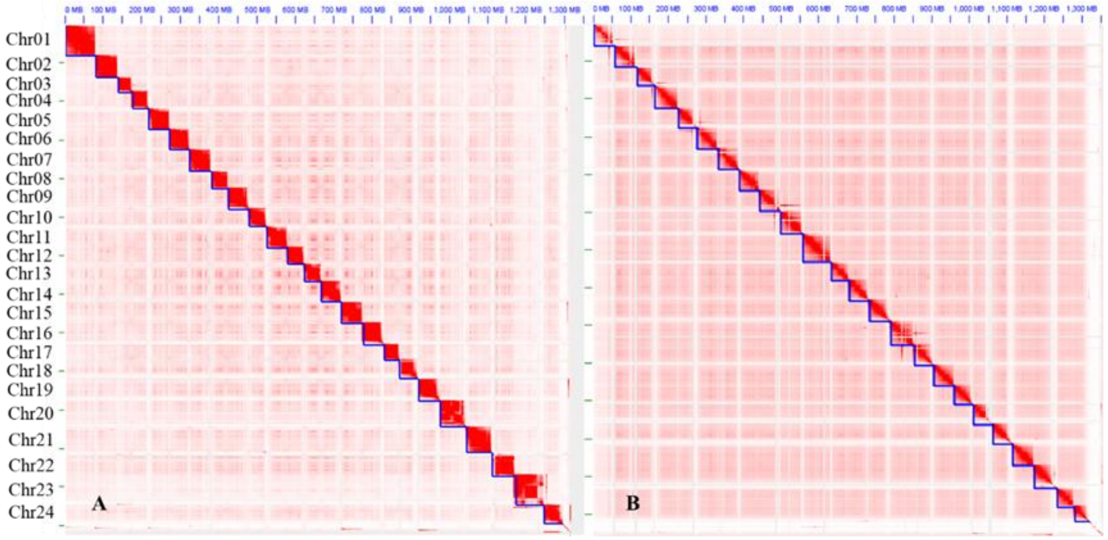

Figure S4 Hi-C interaction heat map of *Harpadon nehereus* (A) and *Saurida undosquamis* (B).

(A)

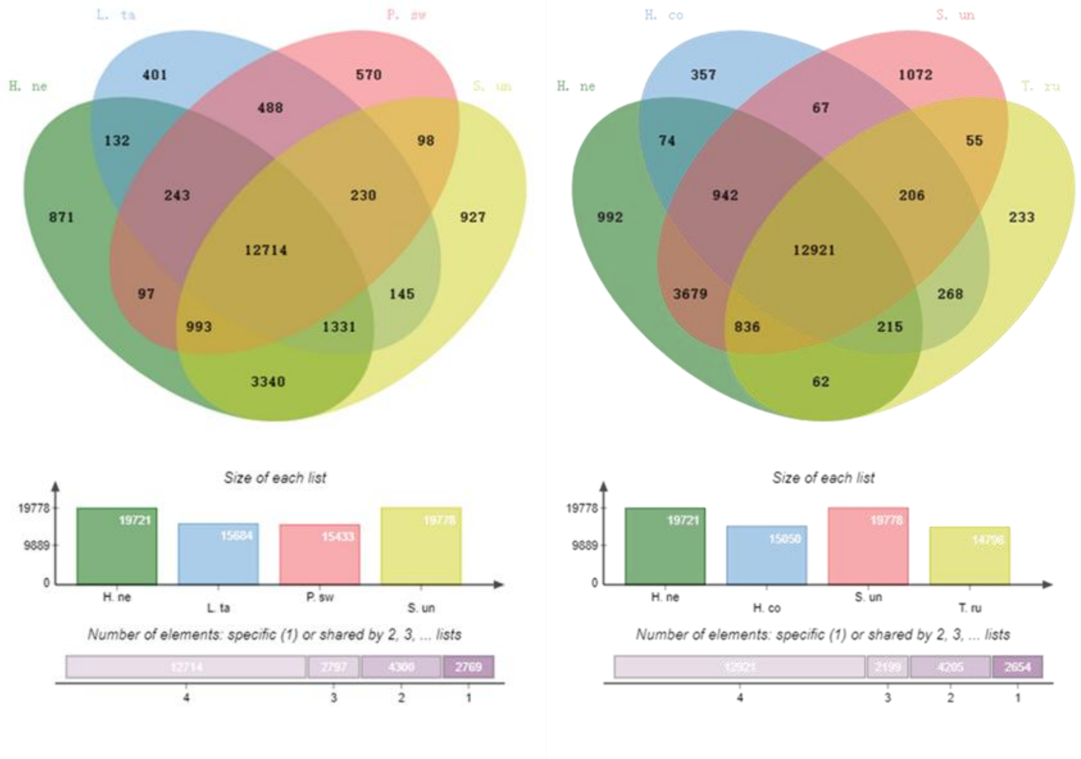
H. ne
L. ta
P. sw
S. un
401
570
488
132
98
871
243
230
927
12714
97
145
993
1331
3340
Size of each list
19778
9889
0
19721
15684
15433
19778
H. ne
L. ta
P. sw
S. un
Number of elements: specific (1) or shared by 2, 3, ... lists
12714
2797
4300
2769
4
3
2
1
H. ne
H. co
S. un
T. ru
357
1072
67
74
55
992
942
206
233
12921
3679
268
836
215
62
Size of each list
19778
9889
0
19721
15050
19778
14796
H. ne
H. co
S. un
T. ru
Number of elements: specific (1) or shared by 2, 3, ... lists
12921
2199
4205
2654
4
3
2
1

(B)

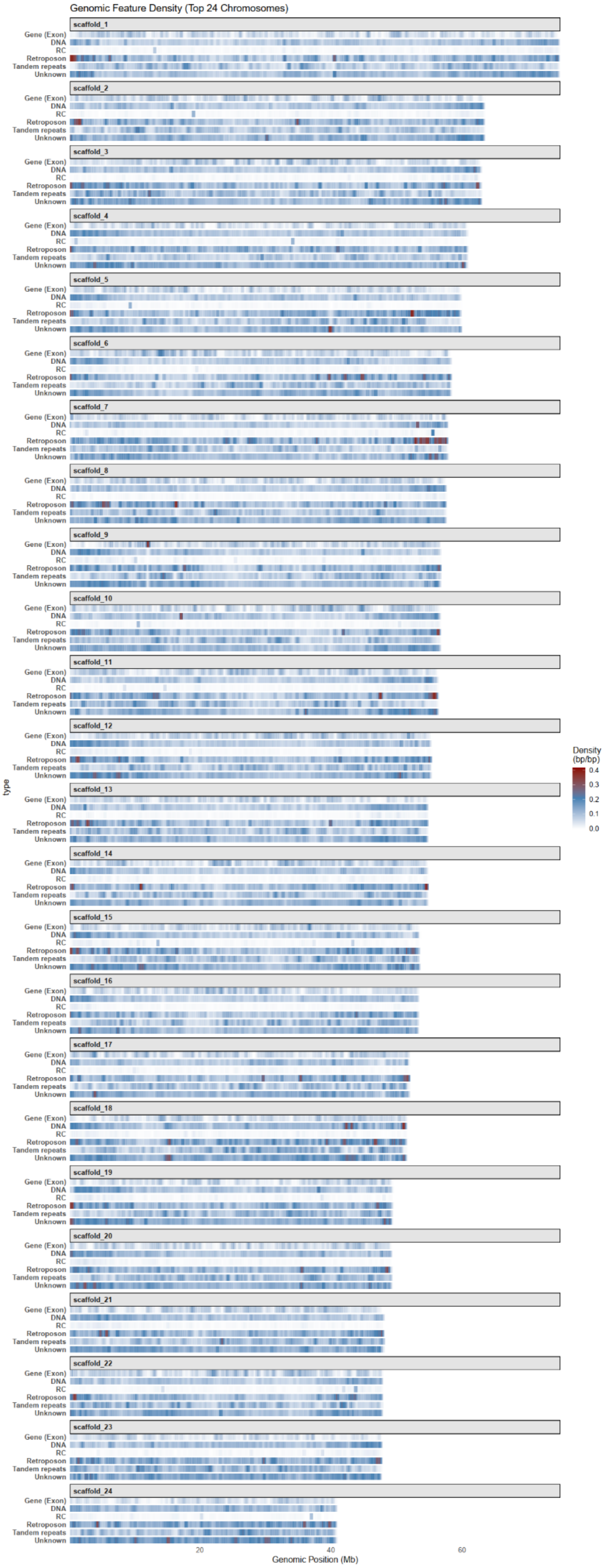
Genomic Feature Density (Top 24 Chromosomes)
scaffold_1
scaffold_2
scaffold_3
scaffold_4
scaffold_5
scaffold_6
scaffold_7
scaffold_8
scaffold_9
scaffold_10
scaffold_11
scaffold_12
scaffold_13
scaffold_14
scaffold_15
scaffold_16
scaffold_17
scaffold_18
scaffold_19
scaffold_20
scaffold_21
scaffold_22
scaffold_23
scaffold_24
Gene (Exon)
DNA
RC
Retroposon
Tandem repeats
Unknown
type
20
40
60
Genomic Position (Mb)
Density (bp/bp)
0.4
0.3
0.2
0.1
0.0

(C)

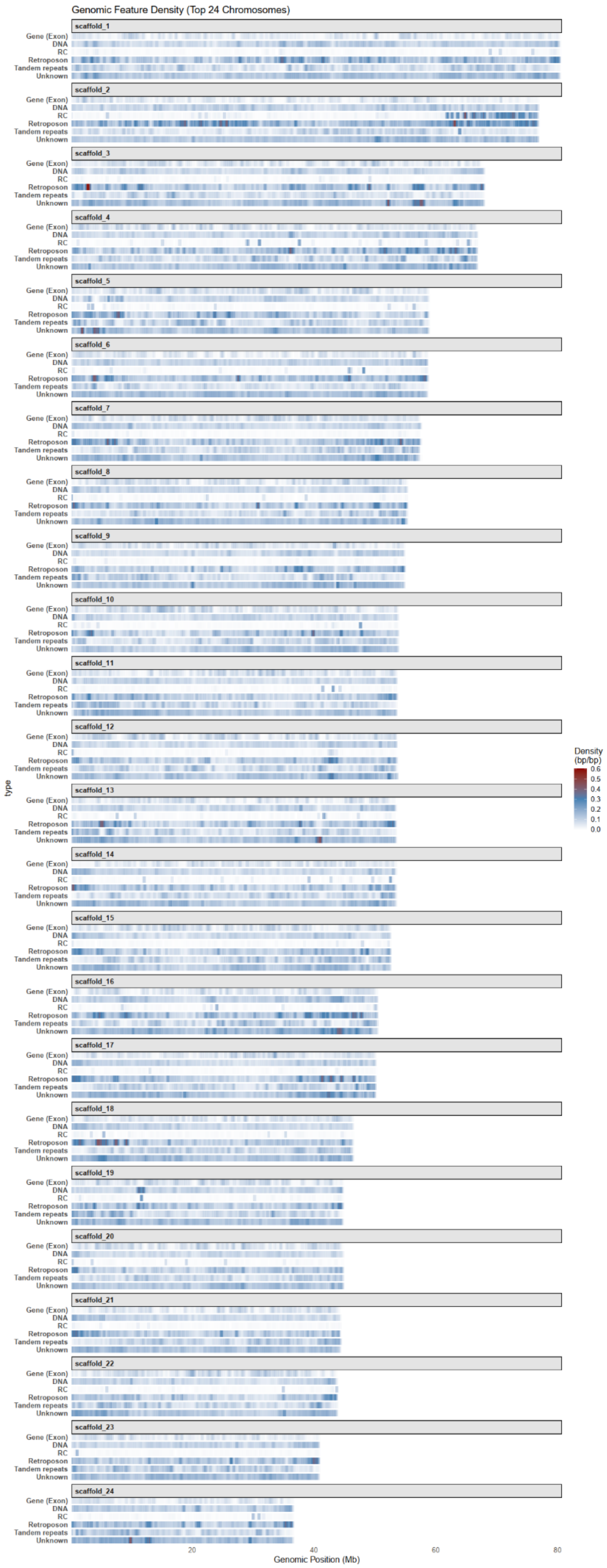
Genomic Feature Density (Top 24 Chromosomes)
scaffold_1
scaffold_2
scaffold_3
scaffold_4
scaffold_5
scaffold_6
scaffold_7
scaffold_8
scaffold_9
scaffold_10
scaffold_11
scaffold_12
scaffold_13
scaffold_14
scaffold_15
scaffold_16
scaffold_17
scaffold_18
scaffold_19
scaffold_20
scaffold_21
scaffold_22
scaffold_23
scaffold_24
Gene (Exon)
DNA
RC
Retroposon
Tandem repeats
Unknown
type
Density (bp/bp)
0.6
0.5
0.4
0.3
0.2
0.1
0.0
20
40
60
80
Genomic Position (Mb)

Figure S5. (A) Statistics of gene family clusters of different species. Sequence densities (bp/bp) of exons, DNA transposons, Rolling-circles, Retroelements, Tandem repeats (small RNAs, satellites, simple repeats, low complexity repeats) and repeats of unknown types of (B) *Harpadon nehereus* and (C) *Saurida undosquamis*. Densities were computed in sliding windows of 500kb with a step size of 10kb.

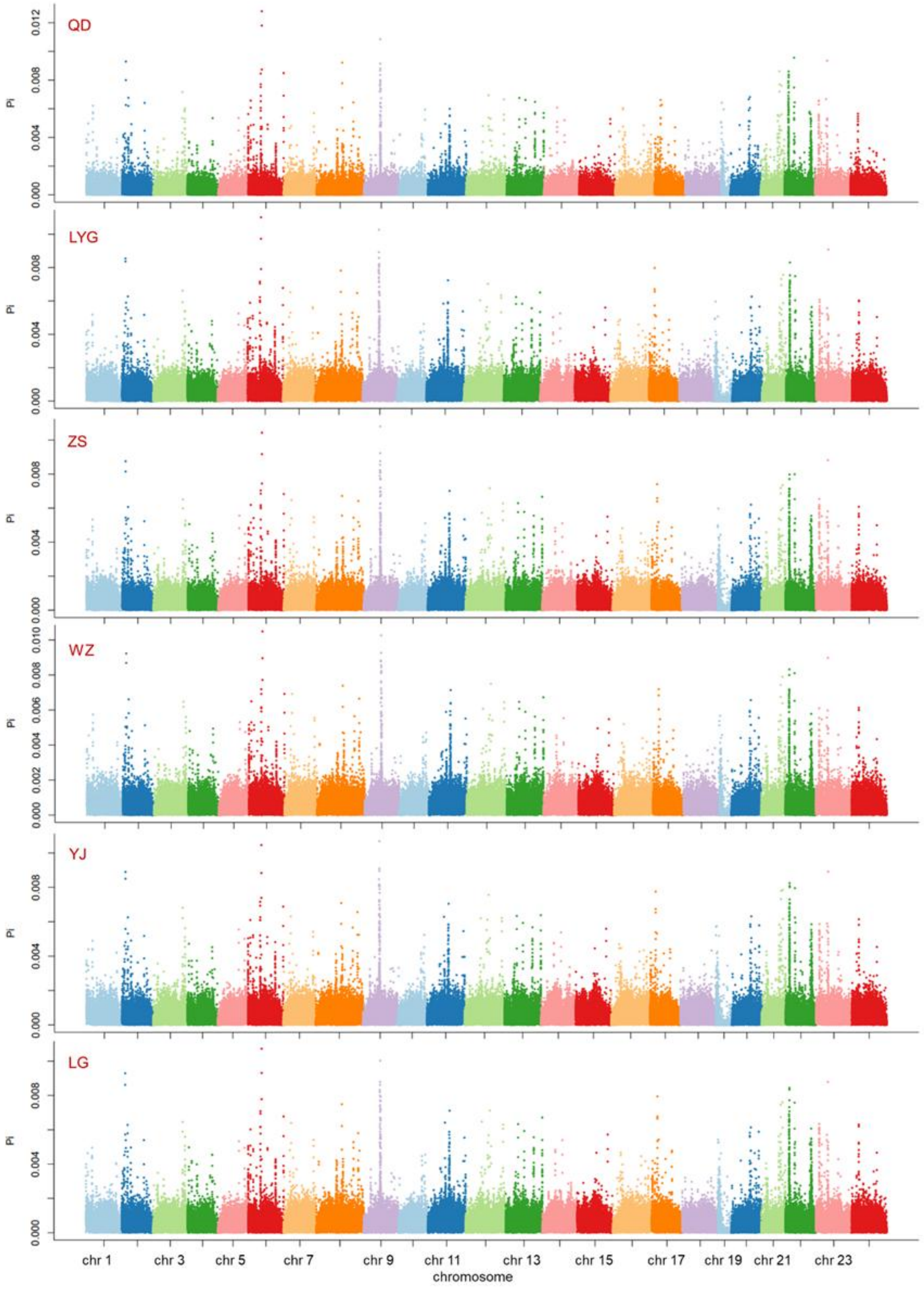

Figure S6. The diagram of genome-wide distribution of nucleotide diversity (π) in 10-kb windows with 5-kb increments.

(A)

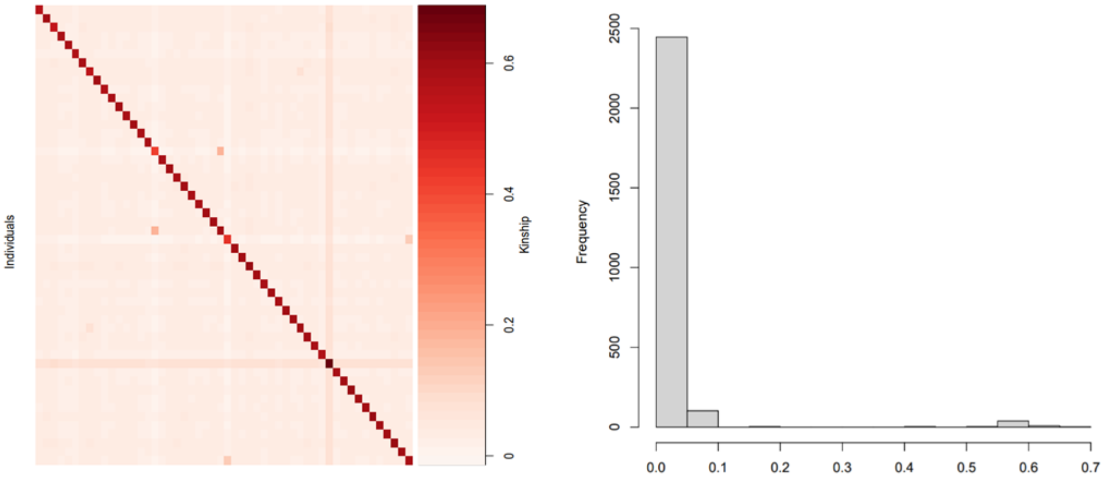

(B)

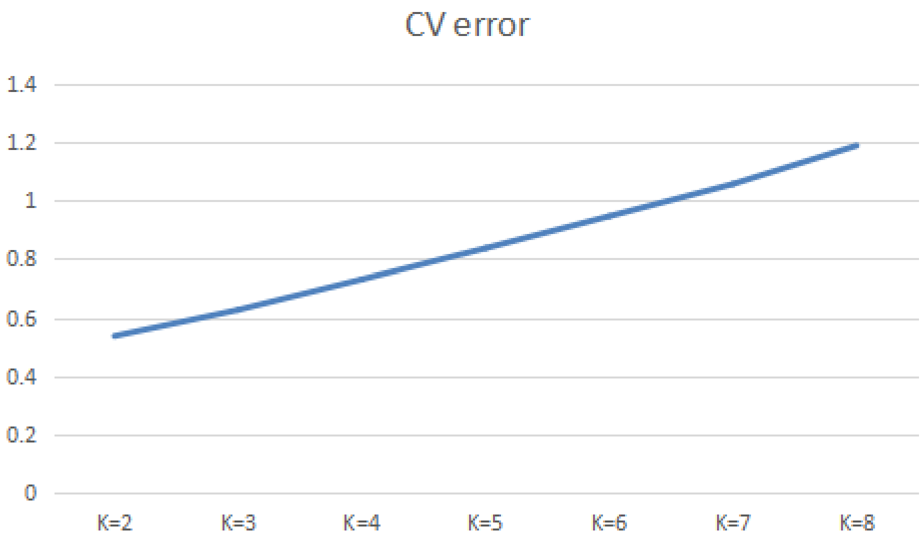

Figure S7. (A) Heatmap of pairwise relatedness coefficients and frequency statistics between *Harpadon nehereus* individuals. (B) Plot of ADMIXTURE cross-validation (CV) error. The CV error scores are shown for K values from 2 to 8. The optimal K value is 2 or 1, as the CV error monotonically increased with K.

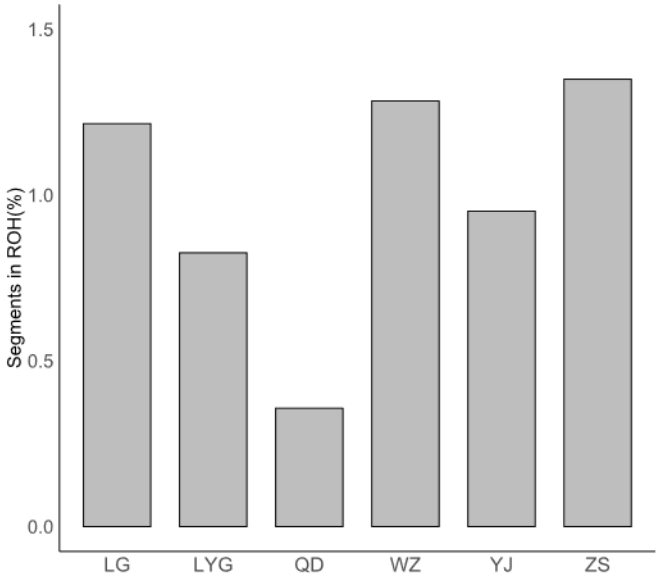

Figure S8. The proportion of ROH segments in the genome of each population of *Harpadon nehereus*.

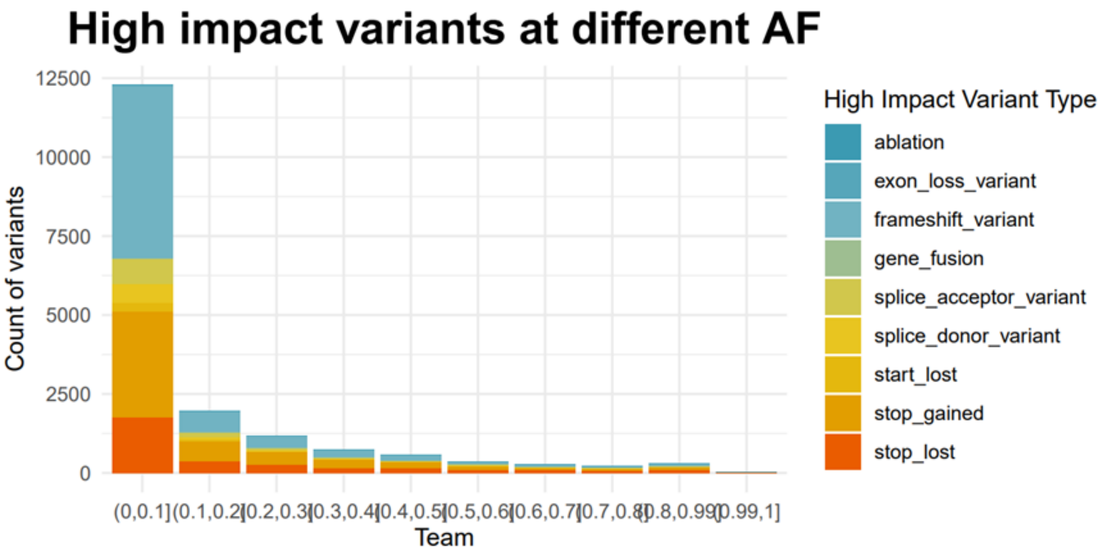

Figure S9. The frequency statistics of different types of high-impact variants in the population of *Harpadon nehereus*.

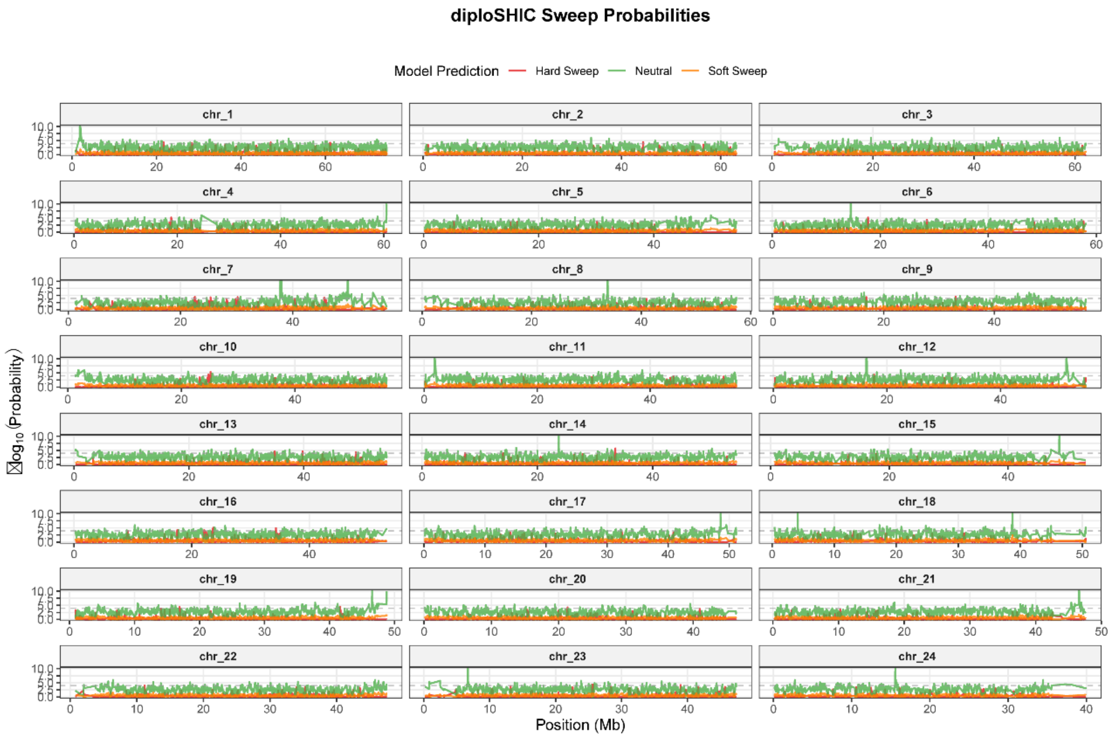

Figure S10. The manhattan plot of diploS/HIC.
