# Supplementary material for: Genomic mechanisms underlying Bombay duck's rapid population surge
Source: Proc Biol Sci. 2026 Nov 4;293(2082):20261106. doi: 10.1098/rspb.2026.1106 (PMC13730804; doi:10.1098/rspb.2026.1106)
Supplement: Supplementary file 1 [file rspb.2026.1106.zip › rspb.2026.1106/rspb20261106_si_003.docx]

Appendix

**Description of key genome assembly statistics**

The BD assembly consisted of 24 pseudochromosomes and 194 unassigned contigs, with a total length of 1,357,870,386bp. The mean scaffold size was 6,228,763.24bp, the largest scaffold was 74,699,408bp. The scaffold N50 of the final chromosome assembly was 55,201,974bp (n=12), N70 was 51,884,348bp (n=17), N90 was 47,791,902bp (n=22). The shortest contig was 5,000bp. The final assembly contained 567 gaps with a total length of 56,700bp. Based on TeloExplorer analysis, 3 pseudochromosomes (2, 10, 22) were assembled with telomere sequences at both ends, 15 pseudochromosomes (1, 4, 6, 8, 9, 11-15, 17-19, 21, 24) had telomere sequences on one end, while telomeres were not recovered for the other six (3, 5, 7, 16, 20, 23). The protein-coding gene annotation contained 44,395 gene models and 49,187 mRNA models, with an average of 1.1 isoforms per gene. Among the annotated mRNAs, 16,858 contained 5’ UTR annotations and 17,539 contained 3’ UTR annotations. Each mRNA contained on average 7.6 exons while each CDS region spanned 7.4 exons. The total length of exons (including isoforms) added up to 93,196,259 bp, or 76,372,894 when only including the longest isoforms. The total length of CDS (including isoforms) summed to 69,994,697 bp, or 59,597,765 when only considering the longest isoforms. The mean CDS length was 1423bp, or ~474 AA. BUSCO analysis revealed that 96.80% complete genes were detected in the BD genome assembly, while 94.7% complete BUSCOs were recovered in the gene annotations (Table S3). A total of 586.99 Mb was identified as repeat elements in the BD genome, accounting for 43.23% of the total genome size, and 8.74% was identified as tandem repeats (Table S4). The rest of the repeats (468.29 Mb, 34.49%) were interspersed repeats mainly consisted of retrotransposons (164.25 Mb, 12.1%) and DNA transposons (124.27 Mb, 9.15%). LTR elements (7.6%) contributed most to the retroelements in the genome, while hobo-Activator (4.36%) was the most highly represented DNA transposon family (Figure S5B).

For the outgroup BL, the estimated genome size was 1198.5 Mb (Figure S3). The assembly consisted of 325 contigs with an N50 of 19.16 Mb. Finally, the contigs were scaffolded into 24 pseudochromosomes (Figure S4, Table S2). The mean scaffold size was 16,921,516.09bp, the largest scaffold was 80,419,649bp. The scaffold N50 of the final chromosome assembly was 53,644,477bp (n=11), N70 was 50,392,151bp (n=16), N90 was 43,787,105bp (n=22). The shortest contig was 6,000bp. The final assembly contained 271 gaps with a total length of 27,100bp. TeloExplorer analysis did not detect telomere sequences at pseudochromosomal ends except for one end of chr17. The protein-coding gene annotation contained 42,089 gene models and 47,044 mRNA models, with an average of 1.1 isoforms per gene. Among the annotated mRNAs, 16,376 contained 5’ UTR annotations and 17,151 contained 3’ UTR annotations. Each mRNA contained on average 7.9 exons while each CDS region spanned 7.8 exons. The total length of exons (including isoforms) added up to 85,854,573 bp, or 69,366,398 when only including the longest isoforms. The total length of CDS (including isoforms) summed to 65,354,096 bp, or 55,241,634 when only considering the longest isoforms. The mean CDS length was 1389, or ~463 AA. BUSCO analysis revealed that 96.70% complete genes were detected in the BL genome assembly, while 94.4% complete BUSCOs were recovered in the gene annotations (Table S3). A total of 611.27 Mb was identified as repeat elements in the BL genome, accounting for 46.32% of the total genome size, and 8.98% was identified as tandem repeats (Table S4). The rest of the repeats (477.39 Mb, 36.17%) were interspersed repeats mainly consisted of retrotransposons (178.13 Mb, 13.5%) and DNA transposons (116.67 Mb, 8.84%). LTR elements (6.7%) contributed most to the retroelements in the genome, while hobo-Activator (3.15%) was the most highly represented DNA transposon family (Figure S5B).
