# Supplementary material for: Genomic mechanisms underlying Bombay duck's rapid population surge
Source: Proc Biol Sci. 2026 Nov 4;293(2082):20261106. doi: 10.1098/rspb.2026.1106 (PMC13730804; doi:10.1098/rspb.2026.1106)
Supplement: Supplementary file 1 [file rspb.2026.1106.zip › rspb.2026.1106/rspb20261106_si_004.docx]

**Supplementary Methods**

**(a) Extraction and sequencing of DNA and RNA**

DNA was extracted from muscle using the phenol/chloroform extraction method. After assessing the DNA quality and concentration using agarose gel electrophoresis and Qubit Fluorometer, the qualified DNA samples were randomly fragmented by Covaris ultrasonic processor, and the pair-end sequencing library with 350 bp insert size was conducted by the steps of end reparation, poly (A) and adaptor addition, purification and PCR amplification, and sequenced by using Illumina Hiseq X-Ten platform in 150 PE mode (Illumina Inc., San Diego, CA, USA). For BD, one 20 kb sequencing SMRTbell library was also constructed from genomic DNA molecules using the BluePippin Size-selection system (Sage Science) and sequenced by PacBio Sequel platform (Pacific Biosciences of California, Menlo Park, CA, USA). For BL, the nanopore libraries were prepared and sequenced using the PromethION DNA sequencer (Oxford Nanopore, Oxford, UK) according to the manufacturer's instructions.

The RNA was extracted using TRIzol Reagent (Invitrogen, USA) and the quality was tested for genome annotation. A Nanodrop ND-1000 spectrophotometer (LabTech, USA) and a 2100 Bioanalyzer (Agilent Technologies, USA) were used to check the transcriptome quality of each tissue. Then, the verified RNA molecules were subsequently pooled equally and reverse transcribed. According to the protocol recommended by the manufacturer, after size selection (cDNA > 4 kb), the SMRTbell library was constructed using amplified cDNA and sequenced by PacBio Sequel platform (Pacific Biosciences of California, Menlo Park, CA, USA).

**(b) Genome evaluation**

Three steps were used to filter the raw data from Illumina sequencing to discard low-quality reads [1] using fastp [2]: 1) reads with adapter sequence were removed; 2) reads with indeterminate base sites’ proportion more than 10% were removed; 3) paired reads with more than 20% low-quality bases (< 5) in either read were removed. The genome size and characteristics were evaluated with sga-preqc (<https://github.com/jts/sga>) by the *K-*mer frequency distribution. The *K*-mer profiles were visualized by GenomeScope.

**(c) Genome assembly and completeness evaluation**

The NextDenovo software (https://github.com/Nextomics/NextDenovo) was used for BD and BL genome assembly. The resulting genome assemblies were corrected with long PacBio and Illumina sequencing reads using NextPolish [3]. Their completeness was evaluated using BUSCO (Benchmarking Universal Single‐Copy Orthologues) [4].

For the chromosome assembly of BD and BL, the Hi-C (High-throughput chromosome conformation capture) technique was applied to capture the interaction information among contigs for clustering and ordering by DNA crosslinking, lysis, chromatin digestion, biotin marking, proximity ligations, crosslinking reversal and purification [5]. Then, the Hi-C library was conducted and sequenced by the Illumina Hiseq X-Ten platform. The Hi-C reads were aligned to the polished genome using BWA (version 0.7.8) [6], and the duplicate and unmapped reads were removed by SAMtools (version 1.3.1) [7]. 3D *de novo* assembly (3D-DNA) pipeline [8], which can anchor the contigs into the chromosomes with an agglomerative hierarchical clustering method, was applied to evaluate the interaction strength and generate the interaction matrix among contigs.

**(d)** **Repeat annotation**

The repetitive sequences consisted of tandem repeats and interspersed repeats (i.e., transposable elements, TEs), which were annotated by homology prediction and *ab initio* prediction. Firstly, we searched repetitive region against RepBase database (http://www.girinst.org/repbase/) [9] using RepeatMasker (version 4.0.6) (http://www.repeatmasker.org/) [10]. Secondly, we constructed a *de novo* repeat library by RepeatModeler (version 1.0.5) (http://www.repeatmasker.org/RepeatModeler.html) [11], and then we searched repetitive sequences against the built repeat library by RepeatMasker. Thus, the repetitive elements were masked in the BD and BL genome for the following gene prediction.

**(e) Gene prediction and functional annotation**

We predicted gene models of the BD and BL genome based on *ab initio*, homology, and RNA-Seq. Augustus (version 3.2.3) [12], was applied for gene *ab initio* prediction. For the homology gene prediction, we downloaded homologous protein sequences including *Chanos chanos*, *Clupea harengus*, *Denticeps clupeoides*, *Salmo trutta*, *Salmo salar*, and *Oncorhynchus mykiss* from Ensembl (http://ensemblgenomes.org/) or NCBI (<http://www.ncbi.nlm.nih.gov/genome/>) and then aligned these proteins against the genome using Diamond [13]. For RNA-seq prediction, the RNA-Seq reads from elven tissues (the heart, eye, brain, gill, kidney, spleen, liver, head-kidney, gonad, stomach, and intestine) were aligned against the genome using STAR (https://github.com/alexdobin/STAR) in splice-aware mode to identify exon-intron boundaries and junction sites. The alignment results were then used by Stringtie (version 1.3.3) [14] for genome-guided transcript assembly, and the resulting transcripts were further aligned back to the genome using minimap2 [15] to provide high-quality structural evidence. Finally, a non-redundant reference gene set was generated by integrating results from *ab initio* prediction, homology searches, and RNA-seq evidence using EvidenceModeler (EVM, v1.1.1) [16] with PASA (version 2.0.1; <https://github.com/PASApipeline/PASApipeline>) employed to refine UTRs and alternative splicing variants.

References

1. Yang X, Liu D, Liu F, Wu J, Zou J, Xiao X, Zhao F, Zhu, B. 2013 HTQC: a fast quality control toolkit for Illumina sequencing data. *BMC Bioinf.* 14, 33. (doi: 10.1186/1471-2105-14-33)
2. Chen S, Zhou Y, Chen Y, Gu J. 2018 Fastp: an ultra-fast all-in-one FASTQ preprocessor. *Bioinformatics* 34, i884-i890. (doi: 10.1093/bioinformatics/bty560)
3. Hu J, Fan J, Sun Z, Liu S. 2020 NextPolish: a fast and efficient genome polishing tool for long-read assembly. *Bioinformatics* 36, 2253-2255. (doi: 10.1093/bioinformatics/btz891)
4. Simao FA, Waterhouse RM, Ioannidis P, Kriventseva EV, Zdobnov EM. 2015 BUSCO: assessing genome assembly and annotation completeness with single-copy orthologs. *Bioinformatics* 31, 3210-3212. (doi: 10.1093/bioinformatics/btv351)
5. Belaghzal H, Dekker J, Gibcus JH. 2017 Hi-C 2.0: An optimized Hi-C procedure for high-resolution genome-wide mapping of chromosome conformation. *Methods* 123, 56-65. (doi: 10.1016/j.ymeth.2017.04.004)
6. Li H. Durbin R. 2009 Fast and accurate short read alignment with Burrows-Wheeler transform. *Bioinformatics* 25, 1754-1760. (doi: 10.1093/bioinformatics/btp324)
7. Li H, Handsaker B, Wysoker A, Fennell T, Ruan J, Homer N, Marth G, Abecasis G, Durbin R. 2009 The Sequence Alignment/Map format and SAMtools. *Bioinformatics* 25, 2078-2079. (doi: 10.1093/bioinformatics/btp352)
8. Dudchenko O. et al. 2017 De novo assembly of the Aedes aegypti genome using Hi-C yields chromosome-length scaffolds. *Science* 356, 92-95. (doi: [10.1126/science.aal3327](https://doi.org/10.1126/science.aal3327))
9. Jurka J, Kapitonov VV, Pavlicek A, Klonowski P, Kohany O, Walichiewicz J. 2005 Repbase Update, a database of eukaryotic repetitive elements. *Cytogenet. Genome Res.* 110, 462-467. (doi: 10.1159/000084979)
10. Bergman CM, Quesneville H. 2007 Discovering and detecting transposable elements in genome sequences. *Brief. Bioinform.* 8, 382-392. (doi: 10.1093/bib/bbm048)
11. Bao Z, Eddy SR. 2002 Automated de novo identification of repeat sequence families in sequenced genomes. *Genome Res.* 12, 1269-1276. (doi: 10.1101/gr.88502)
12. Stanke M, Keller O, Gunduz I, Hayes A, Waack S, Morgenstern B. 2006 AUGUSTUS: ab initio prediction of alternative transcripts. *Nucleic Acids Res.* 34: W435-W439. (doi: 10.1093/nar/gkl200)
13. Buchfink B, Reuter K, Drost H. 2021 Sensitive protein alignments at tree-of-life scale using DIAMOND. *Nat. Methods* 18, 366-368. (doi: 10.1038/s41592-021-01101-x)
14. Pertea M, Pertea GM, Antonescu CM, Chang T, Mendell JT, Salzberg SL. 2015 StringTie enables improved reconstruction of a transcriptome from RNA-seq reads. *Nat. Biotechnol.* 33, 290-295. (doi: 10.1038/nbt.3122)
15. Li H. 2018 Minimap2: pairwise alignment for nucleotide sequences. *Bioinformatics* 34, 3094-3100. (doi: 10.1093/bioinformatics/bty191)
16. Haas BJ, Salzberg SL, Zhu W, Pertea M, Allen JE, Orvis J, White O, Buell CR, Wortman JR. 2008 Automated eukaryotic gene structure annotation using EVidenceModeler and the Program to Assemble Spliced Alignments. *Genome Biol.* 9, R7. (doi: 10.1186/gb-2008-9-1-r7)
